# Correction to: Linking demyelination to compound action potential dispersion with a spike-diffuse-spike approach

**DOI:** 10.1186/s13408-019-0076-1

**Published:** 2019-08-22

**Authors:** Richard Naud, André Longtin

**Affiliations:** 1 Ottawa Brain and Mind Research Institute, Department of Cellular and Molecular Medicine, University of Ottawa, Ottawa, Canada; 2 Department of Physics, University of Ottawa, Ottawa, Canada

Following publication of the original article [1],the authors noticed a mistake in the first paragraph within “Altered propagation”:

The phrase “When an internode undergoes demyelination, its transverse resistance is assumed to **increase** while its capacitance **decreases** [29]” should read: “When an internode undergoes demyelination, its transverse resistance is assumed to **decrease** while its capacitance **increases** [29]”
