# Correction to: Linking demyelination to compound action potential dispersion with a spike-diffuse-spike approach

**DOI:** 10.1186/s13408-020-00083-y

**Published:** 2020-04-20

**Authors:** Richard Naud, André Longtin

**Affiliations:** 1 Ottawa Brain and Mind Research Institute, Department of Cellular and Molecular Medicine, University of Ottawa, Ottawa, Canada; 2 Department of Physics, University of Ottawa, Ottawa, Canada

Following publication of the original article [1], the authors noticed a mistake in the first paragraph within “Altered propagation”:

The phrase “When an internode undergoes demyelination, its transverse resistance is assumed to increase while its capacitance decreases [29]” should read: “When an internode undergoes demyelination, its transverse resistance is assumed to decrease while its capacitance increases [29]”

Figure 1(d) has also been corrected due to an incorrect arrangement of colors:

**Figure 1:**
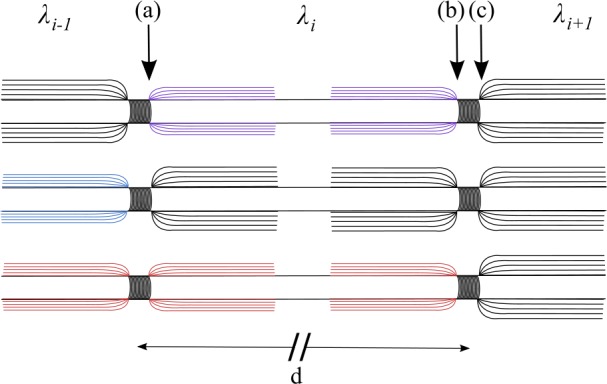
The Stochastic Spike-Diffuse-Spike Model. (**d**) Schematic illustration of two Ranvier nodes separated by a distance d for the three damage configurations. For a propagation from left to right, we consider three possibilities for an action potential starting at node (**a**). Top: demyelination of orthodromic internode. Middle: demyelination of antidromic internode. Bottom: equal demyelination of both anti- and orthodromic internodes
